# Evaluation of Oral Tiazinidine Effects on [intraoperative] Hemodynamic Responses During Direct Laryngoscopy Under General Anesthesia

**DOI:** 10.5812/ircmj.11540

**Published:** 2013-07-05

**Authors:** Masoomeh Tabari, Mohammad Alipour, Hamideh Esalati

**Affiliations:** 1 Department of Anesthesiology, Faculty of Medicine, Mashhad University of Medical Sciences, Mashhad, IR Iran

**Keywords:** Tizanidine, Hemodynamic, Propofol, Premedication

## Abstract

**Background:**

Direct laryngoscopy and tracheal intubation can result in blood pressure and heart rate increase which in turn may lead to myocardial ischemia, cerebral hemorrhage, and even death in susceptible patients. Tizanidine is α_2_-receptor agonists that suppresses central sympathetic system.

**Objectives:**

This study evaluates the effects of oral Tizanidine on hemodynamic responses during operations and aims to determine the appropriate Propofol dosage to maintain anesthesia under BIS monitoring.

**Materials and Methods:**

A double-blind clinical trial has been performed on 70 candidates for elective abdominal surgery undergoing general anesthesia in Educational Hospital of Ghaem, Mashhad, Iran. 35 randomly selected patients (the case group) were given 4 mg of oral Tizanidine 90 minutes before the induction of anesthesia whereas the remaining subjects (the control group) were given placebo. Blood pressure and heart rate before and after induction of anesthesia, and after intubation and extubation, existence of postoperative shivering, and the needed Propofol dosage were measured and recorded. Data analysis was done with T-test and Chi-squared test, using SPSS software version 16.

**Results:**

Variations of blood pressure and heart rate after anesthesia induction, intubation and extubation were less in Tizanidine group generally. Postoperative shivering was reported in 28.6% and 11.4% of patients in control and case group respectively. Average propofol needed dose for anesthesia maintenance in case group was 25% less than the needed amount in the control group.

**Conclusions:**

Using oral Tizanidine as a premedication, yielded stability in blood pressure and heart rate during surgery and decreased required Propofol. Considering its short duration of action, Tizanidine use as a premedication is recommended for sedation and stabilization of hemodynamic responses during the operations.

## 1. Background

Increased blood pressure and heart rate due to laryngoscopy and tracheal intubation may result in myocardial ischemia, cerebral hemorrhage and death during the operations in susceptible patients. Minimizing persistent and severe alterations in heart rate and systemic blood pressure is critical. Increased heart rate raises the myocardial oxygen demand and shortens the diastolic period, which is responsible for maintaining coronary blood flow (1). Myocardial ischemia may occur as a consequence of increased heart rate and blood pressure caused by sympathetic nervous system response during direct laryngoscopy and intubation. Usual recommendations suggest maintaining heart rate and blood pressure at 20% of the basal value (1, 2). α_2_-Receptor agonists decrease the catecholamine’s release; thus, they can diminish the hyper dynamic status which renders the patients prone to ischemia. Mild diminution in heart rate preserves the oxygen supply to demand ratio in an appropriate range. Hemodynamic stabilization, during and after the operations, is the most important mechanism by which the α_2_-receptor agonists reduce peri-operative ischemia (3).

Until now, various drugs have been applied as premedication to reduce severe hemodynamic alteration during anesthesia induction and laryngoscopy. Tizanidine is a α_2_-receptor agonist derived from Clonidine which possesses sedative, anxiolytic and analgesic properties. Its major side-effects include heart rate and blood pressure decrease which are observed less frequently when compared to the side effects of the remaining drugs in this class, such as Clonidine (4). α_2_-agonists have sedative, anxiolytic and analgesic effects and serve to decrease autonomic nervous system responses, i.e., increase in blood pressure and heart rate and the catecholamines release, caused by preoperative anxiousness, laryngoscopy, tracheal intubation and stimulation due to the surgery (5); They are effective in postoperative shivering control as well (6, 7). Administration of Tizanidine may result in hemodynamic stability and decrease the myocardial ischemia and postoperative shivering.

## 2. Objectives

This study attempts to survey the effects of the oral premedication Tizanidine on intraoperative hemodynamic responses and postoperative shivering. It also investigates the required Propofol dosage for anesthesia maintenance of anesthesia.

## 3. Materials and Methods

This double-blind clinical trial surveyed 70 patients aged between 20 to 60 years with the American Society of Anesthesiologists classification (ASA) class 1 and 2 candidates for elective abdominal surgery undergoing general anesthesia at Ghaem Educational, Research and Treatment Center of Mashhad University of Medical Sciences (MUMS). Prior to the surgery all the patients were informed about the investigation and the testimonials were obtained. The patients with positive history of hypertension, Diabetes Mellitus, cardiac disease, renal failure, cirrhosis, neurological disease, or recent consumption of drugs were omitted from the study, as well as the smokers, opiate addicts and the patients with bradycardia (heart rates below 60 beats/min). The study was finally approved in session of 28th may 2011 regional ethics committee, Mashhad University of medical sciences, with code of 89869.

Randomly, the patients were divided into two groups. 90 minutes before the surgery one 4 mg Tizanidine tablet (product of the Swedish company, Novartis^®^) was prescribed to case group, while the other group was treated with placebo. All of the patients underwent basic monitoring including noninvasive observation of blood pressure, electrocardiography, arterial saturation of oxygen (*S*_*a*_*O*_*2*_), heart rate, and bispectral index (BIS) monitoring as they entered the operating room.

Initially, 0.04 mg/kg of Midazolam and 3-4 mcg/kg of Fentanyl were administered as premedication to all patients. Anesthesia induction was performed with 2mg/kg Propofol and 0.6 mg/kg Atracurium. The anesthetics used for maintenance for all patients were 50-150 mcg/kg/h Propofol (which was infused by the pump) alongside with the composition of *N*_*2*_*O* 50% and 50%, as well as 0.02-0.05 mcg/kg/h of Fentanyl, and 0.2 mg/kg of Atracurium per each 20 minutes. Furthermore, Ringer solution was infused in all patients before anesthesia induction. In both groups, the patients with airway insertion difficulties and those for whom the process of laryngoscopy and intubation was prolonged to more than 20 minutes were excluded from the study. BIS was maintained at the level of 40-60 in all patients, thorough out the operation. Provided that BIS fell in the target range, in the cases of severe (more than 20% basic level) increase and decrease in blood pressure either 5-50 mcg/min of nitroglycerin and 5 mg of ephedrine or 500 cc of Ringer solution was infused respectively. Also, 0.5 mg of Atropine was prescribed for cases with heart rates below 40 beats/min.

All of the patients underwent ventilation with tidal volume (TV) and Flow (F) values of 8-10 cc/kg and 10-12/min, respectively. End tidal *CO*_*2*_ (ET* CO*_*2*_) was sustained in a limit of 30-40 mmHg. The patient’s blood pressure and heart rate were monitored and recorded before induction of anesthesia, at two minutes after anesthesia, at one and five minutes after intubation, and after extubation of the airways. Eventually, at the end of the surgery, relaxing effect of anesthetics on skeletal muscles was neutralized using 0.04 mg/kg of Neostigmine and 0.02 mg/kg of Atropine. The amount of consumed Propofol, duration of anesthesia from induction of anesthesia to extubation of the airways, the postoperative shivering presence from patients’ extubation to his/her discharge from the recovery was recorded in a specifically designed checklist. All the observations and measurements were done by one of the authors or under supervision of her. She was completely trained for instruments usage. We used automatic blood pressure device (non-invasive blood pressure) for measuring Blood pressure (mmHg) and pulse oximetry monitor for heart rate (beats/min). We also used biseptical index monitor for anesthesia depth (0-100). All the instruments were calibrated.

Statistical analysis of data was performed by student’s T-test and Chi-squared test via SPSS (Version 16.0). This study aimed to compare instability seen in hemodynamic properties between tizanidine received (case) and non-tizanidine received (control) patients. Regarding the fact that some of the patients would show increase and some other would manifest with decrease in the aforementioned variables, using the Mean in whole sample size do not seem to be appropriate for comparison. So we separated the patients into two groups for statistical analysis: 1- Group with variable reduction and 2- Group with variable increase.

## 4. Results

70 patients (65 males, 5 females) with the mean age of 36.77 ± 11.13 years were enrolled the study in case (33 males, 2 females) and control (32 males, 3 females) groups. The mean age of the patients was reported 36.57 ± 11.31 years in cases and 36.97 ± 11.11 years in controls. The difference between the two groups was not significant. (P = 0.882) Most of the participants were in ASA class I. Only 9 patients (13%) belonged to class II. (5 case patients and 4 controls; P = 0.721). The mean duration of anesthesia was 110.64 ± 44.63 min (35 to 240) in all the patients. Cases (113.57 ± 48.83) and controls (107.71 ± 40.52) were similar in terms of anesthesia duration (P = 0.587).

As demonstrated in Table 1, there was no significant difference of basic and demographic data between the two groups. (P > 0.05) The systolic, diastolic and mean arterial blood pressure together with heart rate average before anesthesia induction was not significantly different between control and case group before the induction of anesthesia (at the start point). Table 2 displays Average percentages of variations in mean arterial pressure (MAP), systolic and diastolic blood pressure, and heart rate in different times. In a 2 minute interval after the anesthesia induction, hemodynamic instability was more prominent in control group. This finding could be seen in both the patients with decrease and those with increase in hemodynamic properties. For example, although the mean diastolic blood pressure changes from the start point was not significantly different between the groups, (-18.28 ± 12.92 mmHg in cases and -24.68 ± 18.32 mmHg for control patients, P = 0.096) the standard deviation was bigger in control patients. In order to achieve a better comparison we considered the changes regarding the decrease or increase in diastolic blood pressure. We found more prominent changes in both the decrease and increase groups among the control patients. (P = 0.006 and P = 0.025 respectively, Table 2). 

**Table 1.** General Demographics and Hemodynamic Parameters Before Anesthesia Induction in the Both Groups

	Control	Case	*P* Value
**Sex**			
Male	3	2	0.643
Female	32	33	
**Age**	36.97 ± 11.11	36.57 ± 11.31	0.882
**BMI (kg/m2)A**	25.92 ± 4.9191.4 ± 92.25	32/4 ± 27.26	0.753
**ASA class**			
I	31	30	0.721
II	4	5	
**Anesthesia duration **	107.71 ± 40.52	113.57 ± 48.83	0.587
**Systolic blood pressure**	128.63 ± 17.61	124.63 ± 13.81	0.294
**Diastolic blood pressure**	84.29 ± 10.98	80.83 ± 10.30	0.179
**Mean arterial pressure**	97.74 ± 13.25	93.77 ± 11.71	0.193
**Heart rate**	92.71 ± 69.16	87.77 ± 11.51	0.154

**Table 2.** Systolic Blood Pressure/Mean Arterial Pressure/ Heart Rate Variation in the Two Groups Based on the Increase and Decrease From the Basal Level During the Course of Time

	Group with variablee reduction			Group with variable increase		
	Control Group	Case group	*P* Value	Control Group	Case Group	*P* Value
**2 minutes after anesthesia induction**						
Systolic blood pressure	-26.05 ± 11.80	-15.81 ± 8.31	< 0.001 ^a^	6.41 ± 2.22	4.30 ± 0	^b^
Mean arterial pressure	-27.92 ± 13.64	-16.31 ± 10.04	< 0.001 ^a^	7.36 ± 8.71	0.77 ± 1.33	0.321
Diastolic blood pressure	-29.32 ± 13.62	-20.25 ± 11.64	0.006 ^a^	11.22 ± 3.50	2.76 ± 3.54	0.025 ^a^
Heart rate	-16.27 ± 10.33	-12.79 ± 6.52	0.177	8.29 ± 9.55	9.98 ± 5.97	0.751
**1 Minutes after intubation**						
Systolic blood pressure	-18/26 ± 9/61	-11.54 ± 7.38	0.008 ^a^	13.64 ± 11.94	9.15 ± 10.95	0.424
Mean arterial pressure	-18/07 ± 9/74	-14.14 ± 10.57	0.194	14.02 ± 12.52	6.94 ± 5.31	0.106
Diastolic blood pressure	-16.87 ± 12.25	-13.33 ± 8.68	0.273	18.63 ± 10.54	9.08 ± 8.81	0.022 ^a^
Heart rate	-14.02 ± 7.51	-74.12 ± 8.30	0.591	15.44 ± 16.85	8.05 ± 7.65	0.184
**5 Minutes after intubation**						
Systolic blood Pressure	-21.79 ± 9.27	-14.55 ± 8.37	0.004 ^a^	8.47 ± 7.54	3.34 ± 3.42	0.169
Mean arterial pressure	-18.06 ± 11.69	-05.16 ± 7.40	0.458	15.17 ± 16.22	2.76 ± 2.68	0.038 ^a^
Diastolic blood pressure	-18.91 ± 11.67	-16.39 ± 9.36	0.398	16.49 ± 9.66	4.01 ± 2.66	0.008 ^a^
Heart rate	-20.14 ± 10.10	-12.95 ± 9.59	0.008 ^a^	10.96 ± 9.33	9.21 ± 5.90	0.683
**1 Minutes after extubation**						
Systolic blood pressure	-7.13 ± 5.10	-9.04 ± 7.84	0.468	19.52 ± 14.54	10.61 ± 8.55	0.017 ^a^
Mean arterial pressure	-8/41 ± 4.44	-10/78 ± 6/32	0.282	22/36 ± 15/63	12/35 ± 12/18	0.020 ^a^
Diastolic blood pressure	-9/86 ± 7/32	11/78 ± 5/66	0.430	23/73 ± 16/90	17/75 ± 19/66	0.308
Heart rate	-22/29 ± 10/03	-14/16 ± 9/21	0.021 ^a^	17/67 ± 14/61	12/90 ± 9/48	0.245

^a^Statistically Significant (P < 0.05)

^b^Cannot be compared due to Subject Scarcity

The findings are summarized with details in Table 2 for systolic and mean blood pressure, and also heart rate. For all the variables, instability is obviously less pronounced in patients who received Tizanidine in. One minute after the intubation, the systolic blood pressure showed 11.54 ± 7.38 mmHg decrease mmHg in case group which is obviously less than the decrease seen in controls (18.26 ± 9.61). (P = 0.008) In patients with blood pressure increase, the changes were also more minute in cases (9.15 ± 10.95 mmHg) compared to controls (13.64 ± 11.49 mmHg). The difference was not statistically significant. (P = 0.424) The situation was similar for diastolic blood pressure, mean arterial blood pressure, and heart rate changes (Table 2). 

In patients with increased mean arterial blood pressure, the changes were more severe in controls (15.17 ± 16.22 mmHg) in comparison to cases following a 5 minute interval after intubation (2.76 ± 2.68 mmHg). The difference was significant. (P = 0.038) In the patients with decrease in hemodynamic properties, the mean arterial blood pressure was found to have -18.06 ± 11.69 mmHg decreases in controls which is obviously more severe than the measured -16.05 ± 7.40 seen in cases. When if the difference was not statistically significant. (P = 0.458) The conditions were similar for systolic, diastolic blood pressure, and heart rate (Table 2). In a 1 minute interval after the extubation, the instability was clearly less intense in patients who received tizanidine. In patients with Heart rate increase, the changes were less severe in cases (12.90 ± 9.48 beats/s) in comparison to controls (17.67 ± 14.61 beats/s). The difference was not statistically significant. (P = 0.245) The Heart rate changes showed -14.16 ± 9.21 beats/s decreases in cases which is significantly less than -22.29 ± 10.03 beats/s seen in controls. (P = 0.021) The findings were similar for systolic, diastolic and mean arterial blood pressure. (Table 2)The frequency distribution for incidence of postoperative shivering in control and case group is shown in Table 3; indicating that 10 subjects (28.6%) in control group and 4 subjects (11.4%) in the case group had postoperative shivering that is considerable, with no statistically significant difference (P = 0.073). Moreover, the required dosage of Propofol for anesthesia maintenance was measured 68.77 ± 16.04 µg/kg/min in case patients against the reported 90.06 ± 17.37 µg/kg/min in controls. As demonstrated in Table 3, the required dosage of Propofol for anesthesia maintenance is significantly higher in the control group patients (P < 0.001). 

**Table 3.** The Incidence of Postoperative Shivering (Chi- square test) and the Mean Required Propofol Dosage (g/kg/min) to Maintain Anesthesia

	Control Group	Case Group	*P* Value
**Postoperative shivering**			
Yes	10 (28.6%)	4 (11.4%)	0.073
No	25 (71.4%)	31 (88.6%)	
**Required propofol dosage**	90.06 ±17.37	68.77 ± 16.04	< 0.001

## 5. Discussion

**Figure 1.**
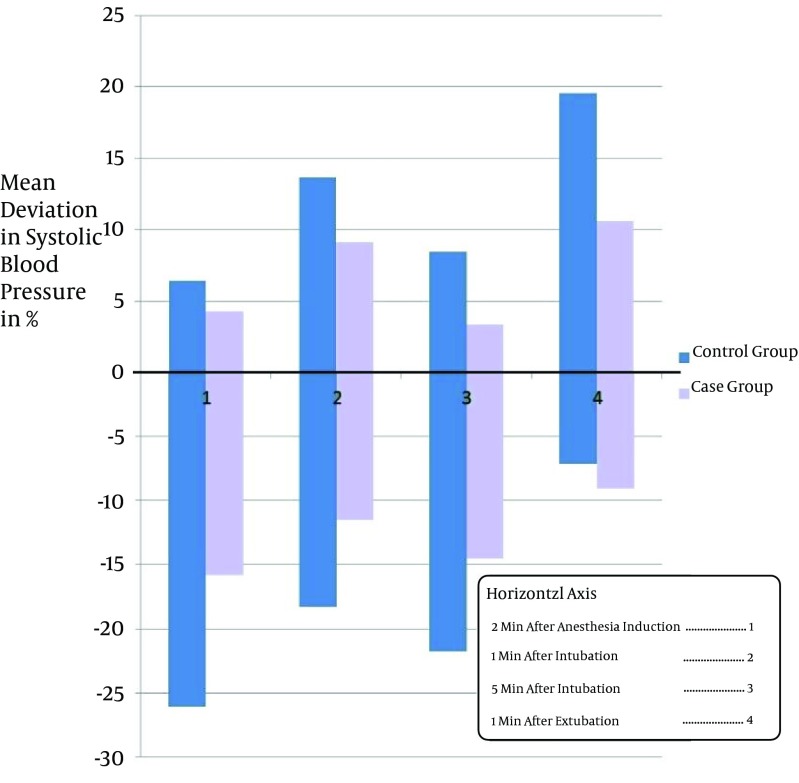
Mean Systolic Blood Pressure Variation in the Two Groups

**Figure 2.**
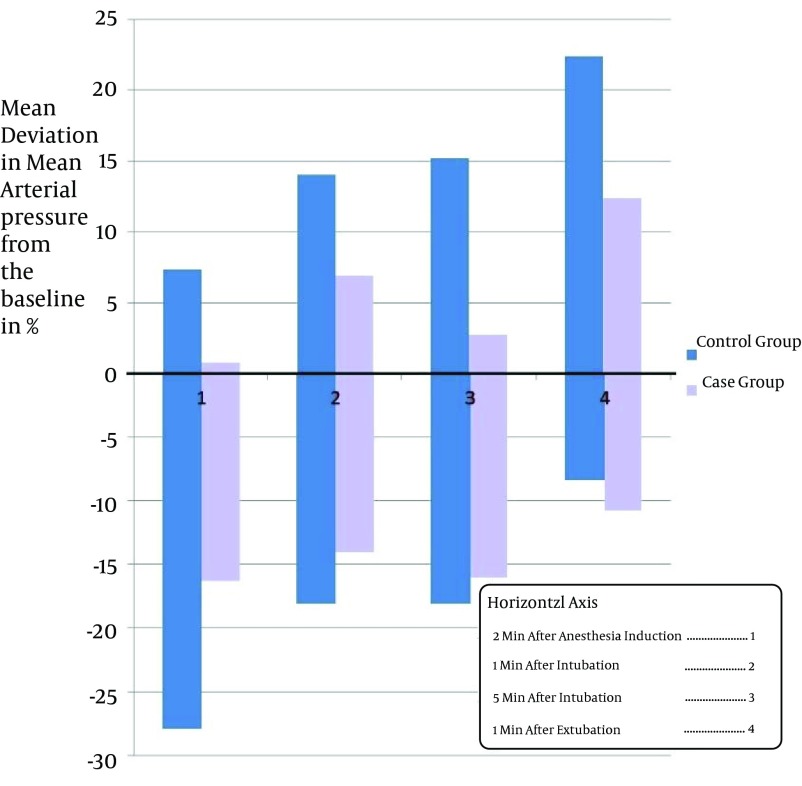
Average of Mean Arterial Pressure Variation in the Two Groups

**Figure 3.**
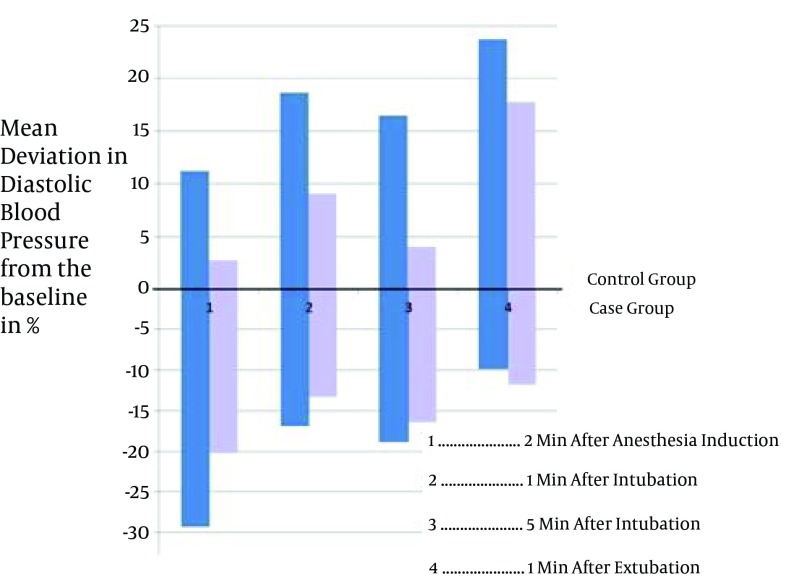
The Mean Diastolic Blood Pressure Variation in the Two Groups

**Figure 4.**
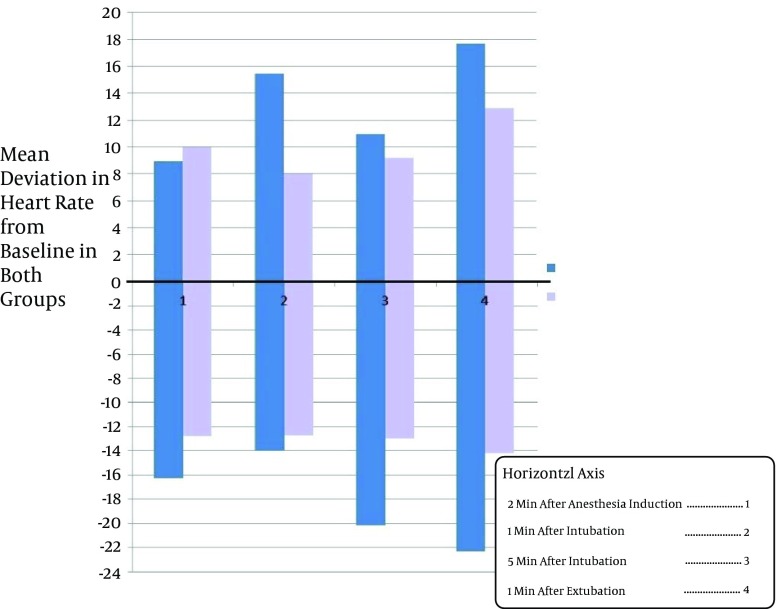
The Mean Heart Rate Variation in the Two Groups

Study of Takenaka et al. suggested that Clonidine can decreases severe alterations in blood pressure and heart rate during the surgeries (8). The idea is supported by other similar studies (9-14). Miettinen et al. showed that Tizanidine can be regarded as an appropriate substitution for Clonidine during the operation because of its shorter duration of action (15). In this study the average percentages of variation in blood pressure, including systolic and diastolic blood pressure and MAP, was below 20% of basal level in all the patients who were given Tizanidine which was mostly less than that of the patients in control group. In some members of control group, blood pressure variation up to 27% of the basal level was recorded (Figures 1-3). A few of the patients had increased blood pressure at 2 minutes after anesthesia induction. This finding may be justifiable by considering the innate differences in responding to the drugs of different individuals. Furthermore, the increase in blood pressure may be an aftereffect of pain caused by propofol infusion, or the anxiety before the surgery. Similarly, variation in heart rate was below 20% basal level in all patients given Tizanidine, whereas the variation of heart rate in all measurements in the control group was higher; even in some cases of control group the heart rate variation surpassed 20% of the basal level (Figure 4). 

Although the prescription of Clonidine was associated with severe decrease of blood pressure in cardiothoracic and non-cardiothoracic surgeries in several studies (16), in our study neither severe hypotension( below 80 mmHg) nor sever bradycardia( below 50 beats/minute) resulted from Tizanidine use and the drug was tolerated with no considerable side-effect, the most common of which being mouth dryness. In addition, the effect of Tizanidine on required propofol dosage for anesthesia maintenance has been surveyed in this study. Average required Propofol dosage to maintain the anesthesia was 92.06 and 68.77 mcg/kg/min in control and case group respectively which showed a statistically significant difference (P < 0.001). In this survey, we found that applying Tizanidine as a premedication caused a 25% diminution in required Propofol for maintenance of anesthesia, which is comparable with previous studies on Clonidine with a reported 20% diminution (17). In accordance to Jafarieh et al., there is not any significantly difference in variations of blood pressure and heart rate between the control group and the case group treated with Clonidine (18).

Incident of postoperative shivering did not show any statistically significant difference between the two groups (P = 0.073); however, we did not find any synonymous studies that evaluated the effect of Tizanidine on postoperative shivering. Pier et al. investigation to compare the effect of Clonidine with Nefopam in preventing postoperative shivering proved that both Clonidine and Nefopam decrease the incidence and severity of postoperative shivering considerably in comparison to placebo, but the recovery time in Clonidine group was significantly more prolonged (19). Butorphanol and Tramadol are more effective than Clonidine to inhibit the shivering, as suggested by Bansal et al. in a study aimed at comparing the effectiveness of these drugs on postoperative shivering (20). Wajima et al. declared that prescribing 4 mg of oral Tizanidine 90 minutes prior to surgery lead to an 18% decline in minimum alveolar concentration (MAC) of Sevoflurane (21). In a study on 60 patients undergoing coronary artery bypass graft (CABG) operation, Manjula et al. concluded that intravenous administration of 4mg Clonidine 30 minutes before the operation diminished the amount of drug required to induce anesthesia (14). Considering this survey and other studies, it can be suggested that low dose (4 mg) of Tizanidine can establish hemodynamic stability. Additionally, it does not have side-effects such as a severe decrease in blood pressure seen in Clonidine. However, more investigations are needed to confirm that using Tizanidine along with lower doses of Propofol during the anesthesia is as safe as the higher doses of Propofol.

Given that cardiovascular status alteration in patients with coronary artery disease (CAD) can lead to dangerous and fatal consequences, we recommend more investigations be carried out to evaluate the effects of Tizanidine in these patients such as the effects of Tizanidine on patients’ recovery time. It is also suggested further studies be performed on effects of various doses of Tizanidine, regarding the effectiveness and cost-effective issues, in comparison with other drugs used to stabilize hemodynamic responses in stressful conditions of the operations (e.g. intubation and extubation of the airways). Prescription of oral Tizanidine as a premedication prior to the operations, in addition to sedative effects, causes stability in blood pressure and heart rate during the operations via decreasing central sympathetic activity. Regarding Tizanidine short duration of action, administration of this drug is recommended for sedating, stabilizing hemodynamic responses during the operations, and for reducing the required dose of anesthetic.
